# A Predominance of Clade 17 *Candida albicans* Isolated From Hemocultures in a Tertiary Care Hospital in Thailand

**DOI:** 10.3389/fmicb.2019.01194

**Published:** 2019-06-14

**Authors:** Linh Thi Truc Pham, Sujiraphong Pharkjaksu, Piriyaporn Chongtrakool, Kamol Suwannakarn, Popchai Ngamskulrungroj

**Affiliations:** Department of Microbiology, Faculty of Medicine Siriraj Hospital, Mahidol University, Bangkok Noi, Thailand

**Keywords:** *Candida albicans*, clade, multilocus sequence typing, virulence factors, antifungal susceptibility, candidemia

## Abstract

*Candida albicans* is one of the most common human fungal pathogens. Candidemia has significant mortality globally. No epidemiological study of *C*. *albicans* based on multilocus sequence typing (MLST) has been conducted in Thailand. Therefore, MLST was used to study the molecular epidemiology of *C*. *albicans* blood strains in a large Thai teaching hospital. *In vitro* virulence phenotypes and antifungal susceptibility testing by broth microdilution were also conducted. Forty-six *C*. *albicans* blood strains from 37 patients were collected from the Department of Microbiology, Siriraj Hospital, in 2016 and 2017. Most patients (71.8%) were more than 60 years old, and the case fatality rate was 54.8%. The male-to-female ratio was 5:3. Thirty-four diploid sequence types (DSTs), including six new DSTs, were identified, with DST2514 (8.7%) and DST2876 (8.7%) as the most common DSTs. Strains were clustered into nine clades. Unlike other studies of *C. albicans* blood strains in Asia, clade 17 was the most common (13 strains, 28.3%). Sequential allelic changes were evident in sequential strains from one patient. All strains produced phospholipase and hemolysin, while none produced proteinase. The ability to form biofilm was found in 82.6% of the strains. Clade 17 strains showed significantly stronger hemolytic activity than non–clade 17 strains (69.2% versus 27.3%; *p* = 0.022). However, no significant association existed between clades and patient mortalities. All were susceptible or wild type to anidulafungin (MIC range = 0.015–0.12 and GM = 0.030), micafungin (MIC range = ≤ 0.008–0.015 and GM = 0.008), caspofungin (MIC range = 0.008–0.12 and GM = 0.036), and amphotericin B (MIC range = 0.25–0.5 and GM = 0.381). Only one strain was resistant to voriconazole (MIC range = ≤ 0.008 to ≥ 8 and GM = 0.010) and fluconazole (MIC range = 0.12–16 and GM = 0.398). In conclusion, a high prevalence of clade 17 *C. albicans* blood strains was found in Thailand, in contrast to other Asian countries. This unique finding might be explained by the strong hemolytic activity that is required for bloodstream infection of *C. albicans.*

## Introduction

*Candida albicans* is a member of the human flora and is commonly colonized in the human digestive tract. *C. albicans* is an important cause of candidiasis (Bougnoux et al., 2006). It can cause infections that range from superficial infections to life-threatening systemic infections. Approximately 75% of all women suffer from vulvovaginal candidiasis at one point in their life span, and *C. albicans* accounts for nearly 90% of such cases (Yazdanparast et al., 2015). *C. albicans* and other *Candida* species including *C. glabrata*, *C. parapsilosis*, *C. tropicalis*, and *C. krusei* can travel through the bloodstream and together are causative agents in 90% of invasive candidiasis cases (Vaezi et al., 2017). Many risk factors contributing to candidiasis were reported, including host-related factors such as immunosuppressive disorders, neutropenia, age, or debilitating diseases, and healthcare-associated factors such as catheter use, surgical interventions, transplantation, or the use of antimicrobial drugs (Yapar, 2014; Diba et al., 2018).

Virulence is defined as the ability of a microbe to damage its host (Casadevall and Pirofski, 1999), and *C. albicans* possesses a wide range of virulence factors, including phenotype switching, adhesin expression, thigmotropism, biofilm formation, and extracellular hydrolytic enzyme secretion, to facilitate tissue invasion and ultimately produce the diseases (Mayer et al., 2013). A recent trend showed increased resistance of non-*albicans Candida* to azole-based antifungal agents, echinocandins, and multiple classes of drugs (Wiederhold, 2017). However, *C. albicans* remains highly susceptible to most antifungal drugs (Bustamante et al., 2014; Posteraro et al., 2015; Doi et al., 2016; Tadec et al., 2016; Tan et al., 2016; Chapman et al., 2017).

Several genotyping methods have been used to study the molecular epidemiology of *C. albicans*. Multilocus sequence typing (MLST) has been widely used to study the molecular characterization of *C. albicans* based on the sequences of seven housekeeping genes (*AAT1a*, *ACC1*, *ADP1*, *MPI1b*, *SYA1*, *VPS13*, and *ZWF1b*) (Bougnoux et al., 2003; Afsarian et al., 2015). This method has identified 19 clades worldwide (Shin et al., 2011; Gong et al., 2018). A 2007 global study of 1,391 *C. albicans* strains showed that clade 1 was the most common (33.6%) (Odds et al., 2007). In Asia, the second and third most common clades were clades 4 (8.8%) and 17 (8.2%) (Odds et al., 2007). When considering only blood strains, a 2016 Korean study of 149 C. *albicans* blood strains showed that clade 18 was the most common (18.8%), followed by clade 4 (15.4%) and clade 1 (14.8%).

So far, no epidemiological study of *C. albicans* based on MLST has been conducted in Thailand. Therefore, MLST was used to study the molecular epidemiology of *C. albicans* strains of candidemia patients at Siriraj Hospital, including virulence factors and antifungal susceptibility profiles.

## Materials and Methods

### Study Sites and Subject Selection

All *C. albicans* strains were collected from positive hemocultures from a diagnostic microbiology laboratory of the Department of Microbiology, Siriraj Hospital, between April 2016 and November 2017. This study was approved by the Siriraj Institutional Review Board, no. SI 091/2016. Informed consent was not required in this work. All strains, collected from the same patients, were considered sequentially collected, as blood samples were taken from different body sites at different time points. Forty-six strains from 37 individual candidemia patients were collected. Available patient records were retrieved from the hospital registry. A healthcare-associated infection was defined as an onset of candidemia symptoms and/or signs after 48 h of admission. As the hospital is a tertiary care referral center, patient origins varied. Most cases (81.25%; 26 of the 32 available patient records) were from central Thailand; 6.25, 6.25, and 6.25% of cases were from Eastern, Northeastern, and Western Thailand, respectively. The case fatality rate was calculated as the ratio of patient deaths to all candidemia cases in this study. All strains were identified using CHROMagar *Candida* chromogenic media (Oxoid, United Kingdom) and RapID^TM^ YEAST PLUS System (Thermo Scientific, United States) followed by single-colony subculture on Sabouraud dextrose agar (SDA; Oxoid, United Kingdom) to prepare pure cultures before performing any further analysis.

### MLST Analysis

**FIGURE 1:**
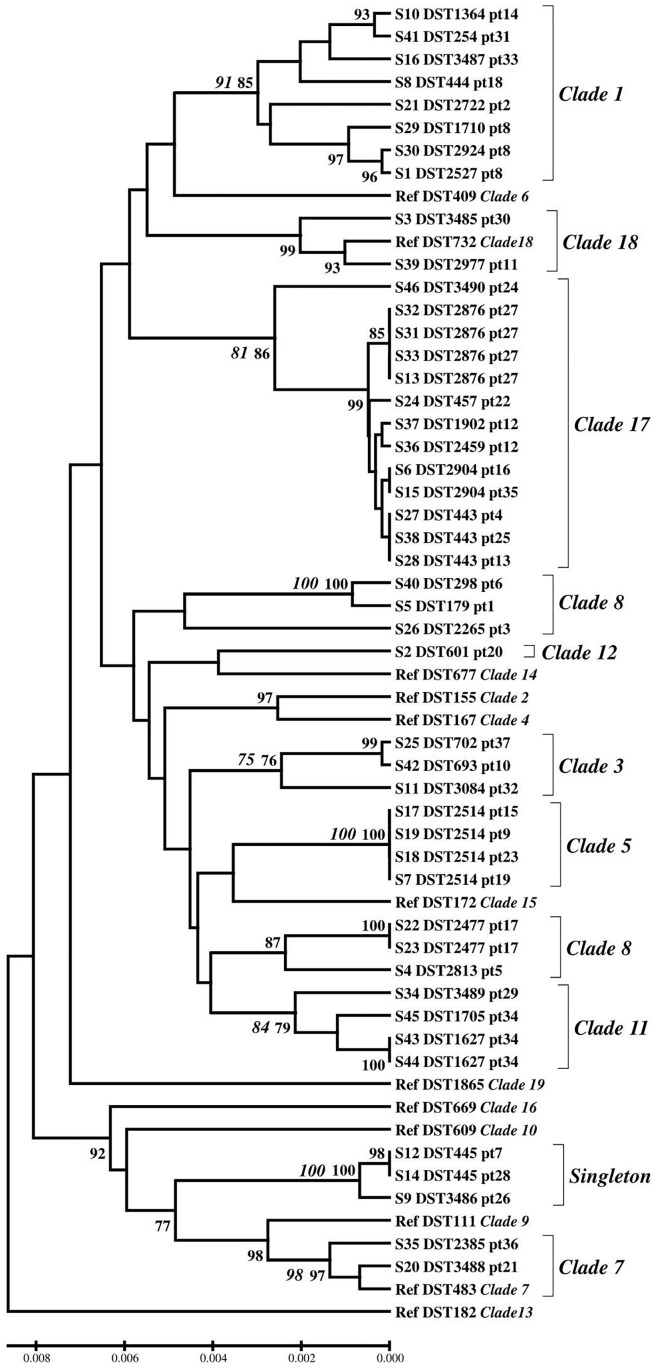
Genetic diversity inferred by UPGMA. Bootstrap support values above 75 for UPGMA (bold) and NJ method (italic bold) were indicated. Ref; reference DST, pt; patient number.

The seven housekeeping genes including *AAT1a*, *ACC1*, *ADP1*, *MPIb, SYA1*, *VPS13*, and *ZWF1b* were used for MLST according to a previously published method (Bougnoux et al., 2002). Briefly, PCR mixtures were prepared in 50 μl reaction volumes containing DNase–RNase free water, 10 × buffer, 25 mM MgCl_2_, 2.5 mM dNTPs, 10 μM for each of the forward and reverse primers of each gene, 0.25 μl of 5 U/μl *Taq* DNA polymerase (Thermo Scientific, United States), and genomic DNA. The condition was set up with a denaturation step at 95°C for 5 min, followed by 35 cycles of 95°C for 40 s, annealing at 50°C for 40 s, extension at 72°C for 1 min, and a final extension step at 72°C for 4 min. Approximately 50 ng of DNA was sequenced using each forward and reverse primer on a DNA analyzer (First Base Company, Singapore). The generated sequences were manually edited using MEGA6 software and aligned using Clustal W. The allelic profile (allele number) for each gene and allele combination (diploid sequence type, DST) for the seven loci of each isolate was assigned, or new DST numbers were obtained from the *C. albicans* MLST database^1^. For cluster analysis, because the base at each polymorphic site can be homozygous or heterozygous, the nucleotide bases were manually edited according to a previous publication (Tavanti et al., 2005). The bases of each taxon were doubled when the site was homozygous and written in pairs when the site was heterozygous. Relationships among concatenated sequences of the seven loci of each strain were determined using cluster analysis using the unweighted pair group method using their arithmetic averages (UPGMA) and the neighbor joining (NJ) method determined by p distance with pairwise deletion of the MEGA 6 software. A bootstrap of 1,000 replications was used for the construction (Shin et al., 2011; Wu et al., 2017). Bootstrap values of ≥75% were defined as statistically significant. Finally, clade numbers were assigned according to previous publications (Odds et al., 2007; Tsai et al., 2015; Wu et al., 2015; Gong et al., 2018) or by using the eBURST method as described previously (Odds et al., 2007). DSTs from clade numbers that were not presented in our strains were also included as reference DSTs for UPGMA and NJ analysis (Figure 1).

### *In vitro* Virulence Study

The test for *C. albicans* phospholipase activity was performed on egg yolk medium according to a previous study (Price et al., 1982). Strains were grown on SDA for 24 h before adjusting to a 0.5 McFarland standard (10^8^ cells/ml). One hundred milliliters of medium containing 13.0 g SDA, 11.7 g NaCl (Merck, Germany) SDA, 11.7 g NaCl (Merck, Germany), 1.48 g CaCl_2_.2H_2_O (Merck, Germany), 2% agar (Oxoid, United Kingdom), and 10% sterile egg yolk (Merck, Germany) was prepared to determine extracellular phospholipase activity (Tsang et al., 2007). Five microliters of a yeast suspension (10^8^ cells/ml) of the tested and control strains was dropped onto the surface of the egg yolk–containing plate and incubated at 37°C for 48 h to measure a precipitation zone around the colony. Extracellular phospholipase activity of *C. albicans* was considered positive when a white precipitation zone was visible around the colony on the plate (Macfarlane and Knight, 1941).

*Candida albicans* strains’ proteinase activity was analyzed on bovine serum albumin medium by the modified Staib method (Staib, 1965; Sachin et al., 2012). Five microliters of a yeast suspension (10^8^ cells/ml) of the tested and control strains was dropped on a medium containing 1.17% yeast carbon base (Becton Dickinson, United States), 0.2% bovine serum albumin (GoldBio, United States), 2% agar (Oxoid, United Kingdom), and 0.01% yeast extract (HiMedia, India) and then incubated at 37°C for 2 days. The presence of a clear halo zone around the colony was recorded as evidence of proteinase activity.

To measure hemolytic activity, a medium was prepared by adding 7 ml fresh sheep blood (Clinag, Thailand) to 100 ml SDA supplemented with 3% of enriched glucose (HiMedia, India) (Luo et al., 2001). Five microliters of a yeast suspension (10^8^ cells/ml) of the tested and control strains was spotted onto the medium. The plate was then incubated at 37°C for 48 h. The presence of a distinct translucent halo indicates positive hemolytic activity.

The ratios of the diameter of the yeast colony to the total diameter of the colony plus the white precipitation zone, the clear halo zone, and the translucent halo zone were used to represent the level of phospholipase, proteinase, and hemolytic activity, respectively (Sachin et al., 2012).

Biofilm formation was determined using the 2,3- bis(2-methoxy-4-nitro-5-sulfophenyl)-2H-tetrazolium-5-carbox anilide (XTT; Thermo Scientific, United States) reduction assay using 96-well microplates as indicated in previous studies (Yigit et al., 2011; Oz et al., 2012). Briefly, yeast cells were adjusted to a 0.5 McFarland standard with yeast extract–peptone–dextrose medium. One hundred microliters of yeast suspension was seeded in 96-well plates and incubated at 37°C for 48 h. The cells were then washed twice with phosphate-buffered saline to remove non-adhering cells. A 50-μl mixture of 1 mg/ml XTT and 10 μl of activation reagent, phenazine methosulfate (HiMedia, India), was added to the wells and further incubated in the dark for 2 h. The biofilms were quantified at A_490_ and defined as low biofilm formation and high biofilm formation by comparing the XTT activity of each strain to the geometrical mean as described previously (Li et al., 2003; Hasan et al., 2009).

Each tested virulence phenotype’s expression levels were classified according to their reference methods (Price et al., 1982; Kantarcioglu and Yucel, 2002; Li et al., 2003; Tsang et al., 2007; Hasan et al., 2009) as follows: zone of activity of phospholipase and proteinase (Price et al., 1982; Kantarcioglu and Yucel, 2002; Tsang et al., 2007): ≤ 0.69 = very strong, 0.70–0.79 = strong, 0.80–0.89 = medium, 0.90–0.99 = weak, and 1.00 = negative; hemolytic activity (Rossoni et al., 2013): < 0.64 = strong positive, 0.64 ≤ to < 1 = positive, and 1.00 = negative; and biofilm formation (Li et al., 2003; Hasan et al., 2009): O.D. value > GM (geometric mean) = high biofilm formation, O.D. value ≤ GM = low biofilm formation, and O.D. value < 0.10 = negative biofilm formation.

### Antifungal Susceptibility Testing

Antifungal susceptibility testing of the *C. albicans* strains was conducted using a commercial microdilution method, *Sensititre*^®^*YeastOne*^®^part YO10 (SYO; Thermo Scientific, United States). The concentrations of antifungal drugs were: anidulafungin 0.015–8 μg/ml, micafungin 0.008–8 μg/ml, caspofungin 0.008–8 μg/ml, amphotericin B 0.12–8 μg/ml, 5-fluorocytosine 0.06–64 μg/ml, posaconazole 0.008–8 μg/ml, voriconazole 0.008–8 μg/ml, itraconazole 0.015–16 μg/ml, and fluconazole 0.12–256 μg/ml, according to the manufacturer’s protocol. *C. krusei* ATCC 6258 and *C. parapsilosis* ATCC 22019 were used as reference strains. Briefly, 20 μl of yeast suspension McFarland standard no. 0.5 (approximately 10^6^ cells/ml) was suspended in 11 ml of YeastOne^®^inoculum broth to give a final working yeast suspension of approximately 1.5–2.5 × 10^3^ cfu/ml. Then, 100 μl of working yeast suspension was added to each well using a multichannel pipetting device. The SYO panels were covered with adhesive seals and incubated at 35°C for 24 h. If the positive control showed no yeast growth, the plate was incubated for an additional 24 h. Minimum inhibitory concentration (MIC) results for all test agents were read according to the manufacturer’s instructions. The criteria used to interpret the epidemiological cutoff values (ECVs) and clinical break points (CBPs) were based on Clinical and Laboratory Standards Institute (CLSI) guidelines: ECVs of anidulafungin = 0.12, micafungin = 0.03, and amphotericin B = 2; CBPs of anidulafungin, micafungin, and caspofungin: S ≤ 0.25, I = 0.5, R ≥ 1; voriconazole: S ≤ 0.12, SDD = 0.25–0.5, R ≥ 1; and fluconazole: S ≤ 2, SDD = 4, R ≥ 8 (Clinical Laboratory Standards Institute, 2012, 2016).

### Statistical Analysis

PASW Statistic 18 (IBM Corporation Armonk, NY, United States) was used for data analysis. To estimate frequencies, descriptive statistical tools were used. GM for biofilm formation was calculated using Microsoft Excel 2013 (Microsoft Corporation, Redmond, WA, United States). Statistical significance of an association between the MLST clusters and virulence phenotype was determined using chi-square or Fisher’s exact test (PASW version 18, IBM, Bangkok, Thailand); *p* ≤ 0.05 was considered statistically significant.

## Results

### Most Candidemia Cases Occurred as Hospital-Associated Infections and in Older Patients

Clinical data were available from 32 of the 37 patients. All patients (100%) had one or more immunocompromised conditions, including receiving immunosuppressants or chemotherapy (10 patients, 31.3%), having diabetes mellitus (10 patients, 31.3%), being a newborn (2 patients, 6.2%), and old age (>60 years old) (23 patients, 71.8%). The case fatality rate was 54.8%. The male-to-female ratio was 5:3 (Supplementary Table S1). Most cases (31 patients, 96.9%) were hospital-associated infections except one who had *Candida* sepsis as a primary diagnosis. A primary source of candidemia was not found in patients in this study.

### MLST Clade 17 and Clade 1 Were Predominant in *C. albicans* Blood Strains in Thailand

The most common allele types of the *AAT1a*, *ACC1*, *ADP1*, *MPIb*, *SYA1*, *VPS13*, and *ZWF1b* genes were allele types 59 (26.1%), 5 (28.3%), 21 (39.1%), 2 (30.4%), 80 (23.9%), 108 (19.6%), and 15 (32.6%), respectively (Supplementary Table S1). The 46 strains yielded 34 distinct DSTs. Forty strains (86.9%) belonged to previously described types (28 DSTs), whereas the remaining six strains (13.0%) belonged to novel DSTs (DST3485, DST3486, DST3487, DST3488, DST3489, and DST3490) that had not previously been identified (Supplementary Table S1). Among the 34 DSTs, the most common DSTs were DST2514 and DST2876 (8.7% for each DST), followed by DST443 (6.5%) and DST1627, DST2477, DST2904, and DST445 (4.4% for each DST). Based on the UPGMA and eBURST data, nine clades and singleton strains were identified. Clade 17 (13 strains, 28.3%) and clade 1 (8 strains, 17.4%) were the most and second most common, respectively (Figure 1 and Supplementary Table S1).

### Sequential Blood Strains Showed Evidence of Sequential Allelic Changes

Five of the 37 patients had sequential strains isolated from their blood. Interestingly, three of these five patients were infected with more than one DST at the same time. Two patients had mixed infections with two DSTs from blood cultures collected on the same date (patient 12 and patient 34; Figure 1 and Supplementary Table S1). Furthermore, *C. albicans* isolates from patient 8 had three different DSTs identified from sequential blood cultures on days 0, 3, and 5 as DST1710, DST2924, and DST2527, respectively (Supplementary Table S1). Further analysis of diploid allele types (DATs) of the three sequential strains showed sequential changes of only one DAT at a time of the two later-collected strains (Table 1). Specifically, DAT7 of the *VPS13* gene of the S29 strain collected on day 0 changed to DAT100 of the S30 strain collected on day 3. Then, DAT5 of the *ADP1* gene of the S29 strain changed to DAT6 of the S1 strain collected on day 5. The profiles of the allelic genes from the patients in whom these different DSTs were detected (patients 8, 12, and 34) are shown in Table 1.

**Table 1:** Genetic positions of polymorphism on allelic genes of the sequential strains from the three patients.

Patient No.	Sample No.	Collection Date	DSTs	ADP1									SYA1	VPS13
	
				815	820	826	889	905	946	985	1005	1012	1623	2359
8	S29	Day 0	1710	A/G										A
	S30	Day 3	2924	A/G										A/T
	S1	Day 5	2527	A										A/T
12	S36	Day 0	2459										C	
	S37	Day 0	1902										A/C	
34	S43/S44	Day 0	1627		C	C	A	A	G	G	T	C		
	S45	Day 0	1705		C/T	C/T	A/G	A/G	A/G	A/G	A/T	C/T		

### Clade 17 Strains Showed Significantly Stronger Hemolytic Activity

As the clade 17 predominance in *C. albicans* blood strains is uncommon, the virulence phenotype and antifungal susceptibility testing results of clade 17 and the other clades were determined and compared. Four virulence phenotypes were tested. First, the phospholipase zone values of the *C. albicans* strains ranged from 0.50 to 0.89. All *C. albicans* strains were phospholipase producers (Table 2 and Supplementary Table S1). Most *C. albicans* strains showed very strong phospholipase activity (30 strains, 65.2%). No weak phospholipase producer was observed. The mean phospholipase activity of all strains was 0.675 (±0.072). Second, none of the *C. albicans* produced proteinase (Supplementary Table S1). Third, hemolytic activity was detected in all strains (Table 2 and Supplementary Table S1). The hemolytic zone values ranged from 0.50 to 0.95. Most strains had positive activity (60.9%). The mean hemolytic activity of the *C. albicans* strains was 0.666 (±0.089). Finally, most *C. albicans* strains (54.3%) were highly able to form a biofilm layer on the bottom surface of each well (Table 2 and Supplementary Table S2). Association analysis using the chi-square test revealed that clade 17 strains showed significantly stronger hemolytic activity than non–clade 17 strains (69.2% versus 37.5% and 24%; *p* = 0.025). There was no significant association between the MLST clades and other virulence phenotypes (*p* > 0.05) (Table 2).

**Table 2:** Number of strains in each MLST cluster in correlation to their phenotypic virulence factors.

	Biofilm formation (%)			Phospholipase activity (%)			Hemolytic activity (%)		Total	Patient outcome^∗∗^ (%)		Total
Clades	High	Low	None	Medium	Strong	Very strong	Positive	Strong positive		Cure	Dead	
1	4 (50)	1 (12.5)	3 (37.5)	8 (100)	2 (25)	6 (75)	5 (62.5)	3 (37.5)	8 (100)	4 (57.1)	3 (42.9)	7 (100)
17	6 (46.2)	4 (30.8)	3 (23)	13 (100)	5 (38.5)	8 (61.5)	4 (30.8)	9 (69.2)	13 (100)	3 (25)	9 (75)	12 (100)
Other^∗^	15 (60)	8 (32)	2 (8)	25 (100)	6 (12)	16 (24)	19 (76)	6 (24)	25 (100)	9 (45)	11 (55)	20 (100)
All strains	25 (54.3)	13 (28.3)	8 (17.4)	46 (100)	13 (28.3)	30 (65.2)	28 (60.9)	18 (39.1)	46 (100)	16 (41)	23 (59)	39 (100)

*P* value	0.335			0.503			0.025		-	0.340		-

*^∗^Clade 3 (n = 3), clade 5 (n = 4), clade 7 (n = 2), clade 8 (n = 6), clade 11 (n = 4), clade 12 (n = 1), clade 18 (n = 2), singleton (n = 3). ^∗∗^Because mixed infections by different DSTs occurred and some strains with identical DSTs from the same patients had variations in virulence phenotypes, all strains from the same patients were considered as different strains and analyzed separately.*

### Clade 17 Showed No Significant Difference in Antifungal Susceptibility Pattern

All strains were susceptible to anidulafungin, micafungin, and caspofungin, and wild type to amphotericin B. Only one isolate (2.2%) was resistant to both voriconazole and fluconazole. The MIC50, MIC90, and geometric mean of all nine drugs are shown in Table 3. There was no significant correlation between clade and antifungal susceptibility. Further association analysis revealed that the clade 17 strains did not cause significantly higher case fatality rates than clade 1 and the other clades (75% versus 42.9% and 55%; *p* = 0.340) (Supplementary Table S2).

**Table 3:** *In vitro* antifungal susceptibilities of *C. albicans* strains from hemoculture*^a,b^*.

Drug	MIC (μ/ml)				No. (%) of strains interpreted by CBPs				No. (%) of strains interpreted by ECVs	
	Range	MIC50	MIC90	GM	S	SDD	I	R	WT	Non-WT
AFG	0.015–0.12	0.015	0.12	0.030	46 (100%)	N/A	0	0	46 (100%)	0
MFG	≤0.008–0.015	≤0.008	0.008	0.008	46 (100%)	N/A	0	0	46 (100%)	0
CFS	0.008–0.12	0.03	0.06	0.036	46 (100%)	N/A	0	0	N/A	N/A
AmB	0.25–0.5	0.5	0.5	0.381	N/A	N/A	N/A	N/A	46 (100%)	0
5-FC	≤0.06–0.25	≤0.06	0.06	0.065	N/A	N/A	N/A	N/A	N/A	N/A
PSO	0.015–≥ 8	0.015	0.03	0.020	N/A	N/A	N/A	N/A	N/A	N/A
VRC	≤0.008–≥ 8	0.008	0.015	0.010	45 (97.83%)	0	N/A	1 (2.17%)	N/A	N/A
ITC	0.03–≥ 16	0.06	0.06	0.052	N/A	N/A	N/A	N/A	N/A	N/A
FLU	0.12–16	0.25	1	0.398	45 (97.83%)	0	N/A	1 (2.17%)	N/A	N/A

*^a^N/A, not applicable; GM, geometric mean; CBP, clinical breakpoint; ECV, epidemiology cut-off value; S, susceptible; SDD, susceptible dose dependent; I, intermediate; R, resistant; WT, wild-type strain; AFG, anidulafungin; MFG, micafungin; CFG, caspofungin; AmB, amphotericin B; 5-FC, 5-flucytosine; PSO, posaconazole; VRC, voriconazole; ITC, itraconazole; FLU, fluconazolea. ^b^Interpretation (CBP and ECV: ug/ml) of susceptibility testing were in accordance with the recommendation of the CLSI for C. albicans; CBPs: Anidulafungin, Micafungin and Caspofungin: S < 25, 1 = 0.5, R > 1; Voriconazole: S < 0.12, SDD = 0.25-0.5, R > 1; Fluconazole: S < 2, SDD = 4, R > 8; ECVs, Anidulafungin = 0.12; Micafungin = 0.03; Amphotericin B = 2.*

## Discussion

In developing countries, candidemia case fatality rates of more than 50% have been reported, which is similar to the 54.8% case fatality rate found in this study (Kaur and Chakrabarti, 2017). Antibiotic resistance is generally one of the common causes of treatment failure of bloodstream infections that leads to patient fatality. However, such resistance is very rare for *C. albicans* blood strains. A comprehensive 2016 study of *C. albicans* bloodstream infections in East and Southeast Asia in found that just 1 out of 309 *C. albicans* blood strains resisted fluconazole, and all strains were sensitive to echinocandins (Tan et al., 2016). Similarly, *C. albicans* blood strains in our study were also highly susceptible to both echinocandins and azoles. This suggests that antifungal resistance is not a cause of treatment failure for *C. albican*s bloodstream infections. Rather, candidemia mortality is associated with patients’ underlying conditions or management such as neutropenia from chemotherapy, septic shock, or ICU admission (Boonyasiri et al., 2013). However, comparisons of these factors for *C. albicans* and non-*albicans Candida* are lacking.

Candidemia risk factors are either healthcare related or host related (Kaur and Chakrabarti, 2017). In this study, almost all patients had both types of risk factors, specifically healthcare-related infections and immunocompromising conditions. In fact, none of the 32 patients whose clinical data were available had no risk factors. However, in-depth details of the candidemia risk factors, including specific healthcare-related factors (such as use of a central vascular catheter, total parenteral nutrition, urinary catheter) and previous colonization of *C. albicans* (Kaur and Chakrabarti, 2017), were not available in our database. With the absence of primary sources regarding candidemia in Thailand, as evidenced by this study (Boonyasiri et al., 2013), determining *C. albicans* colonization is crucial, but it is not common practice in Thailand. Further collection and comprehensive analysis of such data would give an insight into specific risk factors for candidemia in Thailand.

High diversity among sequence types of *C. albicans* was reported in several previous studies (Odds et al., 2007; Shin et al., 2011; Alastruey-Izquierdo et al., 2013; Asadzadeh et al., 2017). Among 46 clinical strains in this study, high sequence variation was also observed with 34 distinct DSTs. DST254 was derived from the parental DST69, the most common DST found using MLST worldwide (Odds et al., 2007). However, DST69 was not found in this study. Additionally, DST443 and DST693, found in 6.5 and 2.2% of cases in this study, respectively, were the most prevalent DSTs in Taiwan (4.6 and 6.7%, respectively) and China (DST443, 7.7%) (Hu et al., 2015; Wang et al., 2015). DST443, DST457, and DST445 are particular sequence types found in Asia (Wang et al., 2015) that were also present in this study.

In general, clade 17 is more common in Asia (8.2%) than other continents (0–6.2%) (Odds et al., 2007). Moreover, a 2015 Chinese study also reported clade 17 as the second most common clade identified from 40 C. *albicans* non-blood strains (Wu et al., 2015). However, when considering only blood strains, clade 17 was rare (3.8%) in the globally collected *C. albicans* samples, and clade 1 was the most common (27.8%) (Odds et al., 2007). Furthermore, no clade 17 strains were found in 149 C. *albicans* blood strains collected in a recent study from Korea (Jung et al., 2016). Only 10.9 and 15% of *C. albicans* blood strains were identified as clade 17 in Taiwan (Wang et al., 2015) and China (Wu et al., 2015), respectively. Therefore, virulence studies were conducted to determine whether the Thai clade 17 isolates were more virulent than isolates from the other clades. The unusually high clade 17 prevalence of the Thai *C. albicans* blood strains might be explained by the stronger hemolytic activity that is required for *C. albicans* bloodstream infection (Furlaneto et al., 2018). However, clade 17 strains did not cause a significantly higher case fatality rate than the other strains. This suggests that many other factors also affect pathogenesis and patient mortality, especially host and environmental factors (Boonyasiri et al., 2013; Kaur and Chakrabarti, 2017).

Phospholipase enzyme plays an important role in host tissue invasion by disrupting the epithelial cell membrane. In general, *C. albicans* strains isolated from blood samples had higher enzyme activity than other anatomical sites (Ibrahim et al., 1995; Mohan Das and Ballal, 2008). For example, blood strains of *C. albicans* from Saudi Arabia (Fotedar and Al-Hedaithy, 2005), the European SENTRY program (Borst and Fluit, 2003), Brazil (Mattei et al., 2013), and Turkey (Atalay et al., 2015) showed 100, 71, 78, and 88% positive phospholipase activity, respectively. Similarly, all *C. albicans* in this study showed positive phospholipase activity. However, despite their similar function regarding host invasion, unlike phospholipase, proteinase activity was typically undetectable in blood strains, whereas 95% of the strains from other sources had proteinase activity (Kantarcioglu and Yucel, 2002). In this study, proteinase activity was undetected in all *C. albicans* strains. Conversely, the European SENTRY program reported that approximately 80% of strains were positive for proteinase (Borst and Fluit, 2003). This suggests that proteinase activity variations might depend not only on the infection source but also on the strains’ geographic location (De Bernardis et al., 2001).

Both hemolysin and biofilm formation are important virulence factors for *C. albicans* (Mayer et al., 2013). Hemolysin facilitates iron acquisition in disseminated candidiasis (Tsang et al., 2007), while biofilm formation protects the yeast from a harsh environment including nutrient storage, metabolic cooperation, and acquisition of new genetic traits (Mayer et al., 2013). In our study, *C. albicans* highly expressed both hemolytic activity (100%) and biofilm formation (82.6%). This suggests that both are important virulence factors for bloodstream infection.

Globally, *C. albicans* showed high susceptibility toward fluconazole, voriconazole, and echinocandins. Azole resistance was generally region dependent and uncommonly found in *C. albicans* (Lyon et al., 2010; Tan et al., 2016). All *C. albicans* strains in this study showed high susceptibility to anidulafungin, micafungin, and caspofungin, similar to previous studies from Australia, the Asia-Pacific, and Italy (Prigitano et al., 2016; Tan et al., 2016; Chapman et al., 2017). Only one (2.17%) *C. albicans* isolate was resistant to voriconazole and fluconazole. This isolate was identified as DST2527, which resisted both voriconazole and fluconazole. This finding contrasts with another study from Taiwan (Wang et al., 2015). Therefore, association of DST2527 with resistance is unlikely. Moreover, when categorizing strains based on ECVs, all *C. albicans* strains also had wild-type MICs for amphotericin B, similar to previous studies (Petlum et al., 2013; Posteraro et al., 2015; Tan et al., 2016; Chapman et al., 2017). These data confirm echinocandins, fluconazole, and amphotericin B as the drugs of choice for treating patients with candidemia (Pappas et al., 2016). Moreover, a recent study showed that a combination of echinocandin and triazole drugs had potent activity for *Candida* spp., and triazole drugs had potent activity for *Candida* spp. (Fakhim et al., 2017). Therefore, antifungal testing for such a combination drug is beneficial, and its interpretation criteria should be established.

## Conclusion

In conclusion, the molecular epidemiology among *C. albicans* blood strains in Thailand showed high genetic diversity. No significant association existed between patient fatality rate and the MLST clades. Clade 17 strains as a predominant clade among isolates showed significant association with hemolytic activity. This finding requires further strain sampling and pathogenetic study.

## Ethics Statement

This study was approved by the Siriraj Institutional Review Board, No. SI 091/2016. Informed consent was not required in this work.

## Author Contributions

PN, PC, and KS designed the study. LP and SP performed the experiments and analyzed the data. LP, SP, and PN wrote the manuscript. All authors read and approved the manuscript.

## Conflict of Interest Statement

The authors declare that the research was conducted in the absence of any commercial or financial relationships that could be construed as a potential conflict of interest.

## Footnotes

1 https://pubmlst.org/calbicans/
