# Supplementary material for: A Predominance of Clade 17 Candida albicans Isolated From Hemocultures in a Tertiary Care Hospital in Thailand
Source: Front Microbiol. 2019 Jun 14;10:1194. doi: 10.3389/fmicb.2019.01194 (PMC6587676; doi:10.3389/fmicb.2019.01194)
Supplement: Supplementary file 1 [file Table_1.DOCX]

**Table S1** Patients' information and diploid sequence types

|  | | | | | | | | | | | | | | | |
| --- | --- | --- | --- | --- | --- | --- | --- | --- | --- | --- | --- | --- | --- | --- | --- |
| **No.** | **Patient** | **Sample** | **Age** | **Gender** | **Collection**  **date** | ***Ward*** | ***Clade*** | ***DST*** | ***AAT1α*** | ***ACC1*** | ***ADP1*** | ***MPIb*** | ***SYA1*** | ***VPS13*** | ***ZWF1b*** |
| 1 | 1 | S5 | U | U | NA | U | 8 | 179 | 25 | 7 | 6 | 3 | 6 | 45 | 47 |
| 2 | 2 | S21 | U | U | NA | U | 1 | 2722 | 2 | 3 | 5 | 2 | 2 | 20 | 12 |
| 3 | 3 | S26 | U | U | NA | U | 8 | 2265 | 4 | 4 | 54 | 9 | 89 | 252 | 105 |
| 4 | 4 | S27 | U | U | NA | U | 17 | 443 | 59 | 5 | 21 | 2 | 80 | 108 | 5 |
| 5 | 5 | S4 | 82 | M | NA | Ward3 | 8 | 2813 | 80 | 10 | 6 | 4 | 30 | 269 | 15 |
| 6 | 6 | S40 | 88 | M | NA | Ward16 | 8 | 298 | 24 | 7 | 6 | 3 | 6 | 27 | 37 |
| 7 | 7 | S12 | 81 | F | NA | Ward8 | S | 445 | 13 | 14 | 12 | 39 | 12 | 110 | 92 |
| ***8*** | ***8*** | ***S29*** | ***66*** | ***M*** | ***0*** | ***Ward12*** | ***1*** | ***1710*** | ***24*** | ***3*** | ***5*** | ***3*** | ***57*** | ***7*** | ***6*** |
| ***9*** | ***8*** | ***S30*** | ***66*** | ***M*** | ***3*** | ***Ward12*** | ***1*** | ***2924*** | ***24*** | ***3*** | ***5*** | ***3*** | ***57*** | ***100*** | ***6*** |
| ***10*** | ***8*** | ***S1*** | ***66*** | ***M*** | ***5*** | ***Ward12*** | ***1*** | ***2527*** | ***24*** | ***3*** | ***6*** | ***3*** | ***57*** | ***100*** | ***6*** |
| 11 | 9 | S19 | 79 | M | NA | Ward9 | 5 | 2514 | 60 | 3 | 4 | 4 | 13 | 260 | 244 |
| 12 | 10 | S42 | 65 | M | NA | Ward6 | 3 | 693 | 1 | 7 | 15 | 6 | 61 | 105 | 112 |
| 13 | 11 | S39 | <1 | F | NA | Ward14 | 18 | 2977 | 5 | 31 | 21 | 34 | 7 | 74 | 5 |
| ***14*** | ***12*** | ***S36*** | ***79*** | ***F*** | ***0*** | ***Ward11*** | ***17*** | ***2459*** | ***59*** | ***5*** | ***21*** | ***2*** | ***67*** | ***108*** | ***15*** |
| ***15*** | ***12*** | ***S37*** | ***79*** | ***F*** | ***0*** | ***Ward11*** | ***17*** | ***1902*** | ***59*** | ***5*** | ***21*** | ***2*** | ***171*** | ***108*** | ***15*** |
| 16 | 13 | S28 | 66 | M | NA | Ward3 | 17 | 443 | 59 | 5 | 21 | 2 | 80 | 108 | 5 |
| 17 | 14 | S10 | <1 | M | NA | ICU1 | 1 | 1364 | 2 | 5 | 2 | 9 | 2 | 6 | 5 |
| 18 | 15 | S17 | 61 | M | NA | Ward13 | 5 | 2514 | 60 | 3 | 4 | 4 | 13 | 260 | 244 |
| 19 | 16 | S6 | 21 | M | NA | Ward11 | 17 | 2904 | 59 | 23 | 21 | 2 | 80 | 108 | 15 |
| ***20*** | ***17*** | ***S22*** | ***46*** | ***F*** | ***0*** | ***Ward10*** | ***8*** | ***2477*** | ***47*** | ***35*** | ***4*** | ***21*** | ***74*** | ***118*** | ***105*** |
| ***21*** | ***17*** | ***S23*** | ***46*** | ***F*** | ***4*** | ***ICU5*** | ***8*** | ***2477*** | ***47*** | ***35*** | ***4*** | ***21*** | ***74*** | ***118*** | ***105*** |
| 22 | 18 | S8 | 30 | F | NA | ICU3 | 1 | 444 | 2 | 5 | 5 | 4 | 2 | 6 | 5 |
| 23 | 19 | S7 | 64 | F | NA | ICU3 | 5 | 2514 | 60 | 3 | 4 | 4 | 13 | 260 | 244 |
| 24 | 20 | S2 | 54 | F | NA | ICU2 | 12 | 601 | 21 | 17 | 21 | 19 | 27 | 83 | 22 |
| 25 | 21 | S20 | 15 | F | NA | Ward7 | 7 | 3488 | 6 | 3 | 10 | 9 | 38 | 46 | 12 |
| 26 | 22 | S24 | 67 | F | NA | Ward8 | 17 | 457 | 53 | 5 | 21 | 2 | 80 | 108 | 15 |
| 27 | 23 | S18 | 66 | M | NA | Ward13 | 5 | 2514 | 60 | 3 | 4 | 4 | 13 | 260 | 244 |
| 28 | 24 | S46 | 71 | M | NA | Ward1 | 17 | 3490 | 4 | 3 | 21 | 4 | 67 | 295 | 29 |
| 29 | 25 | S38 | 74 | M | NA | ICU4 | 17 | 443 | 59 | 5 | 21 | 2 | 80 | 108 | 5 |
| 30 | 26 | S9 | 74 | M | NA | Ward15 | S | 3486 | 28 | 3 | 12 | 39 | 12 | 110 | 92 |
| ***31*** | ***27*** | ***S13*** | ***88*** | ***M*** | ***0*** | ***ICU2*** | ***17*** | ***2876*** | ***59*** | ***5*** | ***21*** | ***2*** | ***80*** | ***20*** | ***15*** |
| ***32*** | ***27*** | ***S31*** | ***88*** | ***M*** | ***0*** | ***ICU2*** | ***17*** | ***2876*** | ***59*** | ***5*** | ***21*** | ***2*** | ***80*** | ***20*** | ***15*** |
| ***33*** | ***27*** | ***S32*** | ***88*** | ***M*** | ***0*** | ***ICU2*** | ***17*** | ***2876*** | ***59*** | ***5*** | ***21*** | ***2*** | ***80*** | ***20*** | ***15*** |
| ***34*** | ***27*** | ***S33*** | ***88*** | ***M*** | ***0*** | ***ICU2*** | ***17*** | ***2876*** | ***59*** | ***5*** | ***21*** | ***2*** | ***80*** | ***20*** | ***15*** |
| 35 | 28 | S14 | 86 | M | NA | Ward4 | S | 445 | 13 | 14 | 12 | 39 | 12 | 110 | 92 |
| 36 | 29 | S34 | 74 | M | NA | ICU5 | 11 | 3489 | 60 | 27 | 21 | 1 | 75 | 11 | 15 |
| 37 | 30 | S3 | 80 | M | NA | Ward2 | 18 | 3485 | 5 | 32 | 6 | 34 | 7 | 13 | 20 |
| 38 | 31 | S41 | 18 | M | NA | ICU5 | 1 | 254 | 2 | 2 | 5 | 9 | 2 | 6 | 5 |
| 39 | 32 | S11 | 59 | M | NA | ICU5 | 3 | 3084 | 62 | 12 | 21 | 1 | 6 | 59 | 72 |
| 40 | 33 | S16 | 52 | M | NA | ICU4 | 1 | 3487 | 23 | 3 | 5 | 9 | 2 | 21 | 12 |
| ***41*** | ***34*** | ***S43*** | ***80*** | ***F*** | ***0*** | ***Ward11*** | ***11*** | ***1627*** | ***60*** | ***13*** | ***6*** | ***1*** | ***7*** | ***11*** | ***15*** |
| ***42*** | ***34*** | ***S44*** | ***80*** | ***F*** | ***0*** | ***Ward11*** | ***11*** | ***1627*** | ***60*** | ***13*** | ***6*** | ***1*** | ***7*** | ***11*** | ***15*** |
| ***43*** | ***34*** | ***S45*** | ***80*** | ***F*** | ***0*** | ***Ward11*** | ***11*** | ***1705*** | ***60*** | ***13*** | ***21*** | ***1*** | ***7*** | ***11*** | ***15*** |
| 44 | 35 | S15 | 64 | F | NA | ICU4 | 17 | 2904 | 59 | 23 | 21 | 2 | 80 | 108 | 15 |
| 45 | 36 | S35 | 79 | F | NA | Ward12 | 7 | 2385 | 59 | 5 | 10 | 2 | 80 | 108 | 15 |
| 46 | 37 | S25 | 92 | F | NA | Ward5 | 3 | 702 | 1 | 7 | 15 | 24 | 61 | 105 | 8 |
| U= unknown, NA= not applicable | | | | | | | | | | | | | | | |

**Table S2** Diploid sequence types and *in vitro* virulence phenotypes

| Isolate | DST*^*^* | Clade | Level of *in vitro* virulence phenotypes | | | | Patient  outcome |
| --- | --- | --- | --- | --- | --- | --- | --- |
|  |  |  | **Phospholipase** | **Proteinase** | **Hemolytic activity** | **Biofilm formation** |  |
| S1 | 2527 | 1 | VS | N | SP | NBF | Dead |
| S2 | 601 | 12 | VS | N | SP | LBF | Dead |
| S3 | ***3485*** | 18 | VS | N | SP | HBF | Dead |
| S4 | 2813 | 8 | VS | N | P | NBF | Dead |
| S5 | 179 | 8 | S | N | SP | LBF | Unknown |
| S6 | 2904 | 17 | VS | N | SP | HBF | Dead |
| S7 | 2514 | 5 | VS | N | P | LBF | Cure |
| S8 | 444 | 1 | VS | N | P | HBF | Cure |
| S9 | ***3486*** | S | VS | N | P | HBF | Cure |
| S10 | 1364 | 1 | S | N | P | HBF | Dead |
| S11 | 3084 | 3 | VS | N | P | HBF | Cure |
| S12 | 445 | S | VS | N | SP | HBF | Dead |
| S13 | 2876 | 17 | VS | N | P | NBF | Cure |
| S14 | 445 | S | VS | N | P | HBF | Dead |
| S15 | 2904 | 17 | S | N | SP | LBF | Dead |
| S16 | ***3487*** | 1 | VS | N | P | NBF | Cure |
| S17 | 2514 | 5 | VS | N | SP | HBF | Cure |
| S18 | 2514 | 5 | S | N | P | LBF | Cure |
| S19 | 2514 | 5 | VS | N | P | HBF | Cure |
| S20 | ***3488*** | 7 | VS | N | P | LBF | Cure |
| S21 | 2722 | 1 | VS | N | P | NBF | Unknown |
| S22 | 2477 | 8 | VS | N | P | LBF | Unknown |
| S23 | 2477 | 8 | VS | N | P | LBF | Unknown |
| S24 | 457 | 17 | VS | N | SP | HBF | Cure |
| S25 | 702 | 3 | VS | N | P | HBF | Dead |
| S26 | 2265 | 8 | VS | N | SP | HBF | Unknown |
| S27 | 443 | 17 | VS | N | P | HBF | Unknown |
| S28 | 443 | 17 | VS | N | P | LBF | Cure |
| S29 | 1710 | 1 | VS | N | SP | LBF | Dead |
| S30 | 2924 | 1 | VS | N | SP | HBF | Dead |
| S31 | 2876 | 17 | VS | N | SP | NBF | Cure |
| S32 | 2876 | 17 | S | N | P | LBF | Cure |
| S33 | 2876 | 17 | VS | N | SP | NBF | Cure |
| S34 | ***3489*** | 11 | M | N | P | NBF | Cure |
| S35 | 2385 | 7 | S | N | P | LBF | Dead |
| S36 | 2459 | 17 | S | N | SP | LBF | Cure |
| S37 | 1902 | 17 | S | N | SP | HBF | Cure |
| S38 | 443 | 17 | VS | N | SP | HBF | Cure |
| S39 | 2977 | 18 | M | N | P | HBF | Dead |
| S40 | 298 | 8 | M | N | P | HBF | Dead |
| S41 | 254 | 1 | S | N | P | HBF | Cure |
| S42 | 693 | 3 | VS | N | P | HBF | Unknown |
| S43 | 1627 | 11 | S | N | P | HBF | Cure |
| S44 | 1627 | 11 | S | N | P | HBF | Cure |
| S45 | 1705 | 11 | S | N | P | HBF | Cure |
| S46 | ***3490*** | 17 | S | N | SP | HBF | Dead |

*^*^*MLST-based DST, multi locus sequence type-based diploid sequence type; new DSTs detected in this study are shown in italicized bold; VS (very strong), S (strong), M (medium), W (weak), N (negative), SP (strong positive), P (positive), HBF (high biofilm formation), LBF (low biofilm formation), NBF (negative biofilm formation).
